# A Biomimetic Heparinized Composite Silk-Based Vascular Scaffold with sustained Antithrombogenicity

**DOI:** 10.1038/s41598-017-04510-1

**Published:** 2017-06-30

**Authors:** Masoud Zamani, Mona Khafaji, Mohammad Naji, Manouchehr Vossoughi, Iran Alemzadeh, Nooshin Haghighipour

**Affiliations:** 1 Department of Chemical and Petroleum Engineering, Sharif University of Technology, Tehran, Iran; 2 Institute for Nano-science and Nano-technology, Sharif University of Technology, Tehran, Iran; 3 Department of Anatomy, School of Medicine, Tehran University of Medical Sciences, Tehran, Iran; 4 National Cell Bank of Iran, Pasteur Institute of Iran, Tehran, Iran

## Abstract

Autologous grafts, as the gold standard for vascular bypass procedures, associated with several problems that limit their usability, so tissue engineered vessels have been the subject of an increasing number of works. Nevertheless, gathering all of the desired characteristics of vascular scaffolds in the same construct has been a big challenge for scientists. Herein, a composite silk-based vascular scaffold (CSVS) was proposed to consider all the mechanical, structural and biological requirements of a small-diameter vascular scaffold. The scaffold’s lumen composed of braided silk fiber-reinforced silk fibroin (SF) sponge covalently heparinized (H-CSVS) using Hydroxy-Iron Complexes (HICs) as linkers. The highly porous SF external layer with pores above 60 μm was obtained by lyophilization. Silk fibers were fully embedded in scaffold’s wall with no delamination. The H-CSVS exhibited much higher burst pressure and suture retention strength than native vessels while comparable elastic modulus and compliance. H-CSVSs presented milder hemolysis *in vitro* and significant calcification resistance in subcutaneous implantation compared to non-heparinized ones. The *in vitro* antithrombogenic activity was sustained for over 12 weeks. The cytocompatibility was approved using endothelial cells (ECs) and vascular smooth muscle cells (SMCs) *in vitro*. Therefore, H-CSVS demonstrates a promising candidate for engineering of small-diameter vessels.

## Introduction

Cardiovascular disease is the leading global cause of death with over 45% of those related to coronary artery disease^1^. Although minimally invasive treatments such as balloon angioplasty and stenting can be used in some cases, the most widely used therapeutic procedure for high-risk patients remains the coronary artery bypass graft^2^. Clinically, the current “gold standard” is autologous grafting, where the saphenous vein and an internal mammary artery with excellent long-term patency are utilized to bypass the diseased vessel^3, 4^. However, the use of autologous grafts is associated with problems such as accelerated atherosclerotic changes (limited to veins), donor site morbidity and the need for additional harvest. On the other hand, some issues such as previous harvest and accentuated general vascular disease are the reasons why the use of autografts is limited for many patients^5, 6^. Hence, there is a huge demand to develop a functional alternative to autologous grafting. Currently available synthetic vascular prostheses such as expanded polytetrafluoroethylene (PTFE, Teflon^®^) and polyethylene terephthalate (PET, Dacron^®^) have not resulted in satisfactory outcomes in small-diameter vascular replacement because of the poor biocompatibility at the blood–biomaterial interface which causes thrombosis. Moreover, anastomotic intimal hyperplasia is usually occurred due to the compliance mismatch between the prosthesis and native tissue^7, 8^. The latter and also some other material-related issues such as stenosis, thromboembolization, calcium deposition, and infection are proved to be the cause of failure of small-diameter (<6 mm) prosthetic vascular grafts in the long term^9^. In addition, in the pediatric population, the lack of growth potential of the synthetic vascular grafts requires multiple operations as a child grows^10^.

Therefore, the only remaining solution to address the need for small-diameter vascular grafts is tissue engineering approaches, i.e. creation of a functional blood vessel by the combination of a biodegradable scaffold with autologous vascular cells^11, 12^. In this regard, the scaffold plays a critical role in the regeneration process since,as a temporary three-dimensional template, it should not only provide structural, mechanical and biological support but also guide and orient the process of tissue formation and remodeling. In order to achieve a functional blood vessel with three main cell layers (i.e. intima, media, and adventitia)^13^, the small-diameter vascular scaffold should have certain properties, including good biocompatibility, suitable mechanical properties (i.e. suitable flexibility, compliance matching that of native arteries suture retention strength and resistance to aneurysm formation), appropriate porosity for each vascular cells, degradation rate relative to extracellular matrix (ECM) deposition and very low thrombogenicity^14, 15^.

In general, natural biomaterials possess favorite biocompatibility while synthetic polymers can provide suitable mechanical properties. However, the combination of biocompatibility and mechanical properties is difficult to achieve with a homogeneous construct. To overcome this issue, multilayered grafts composed of different synthetic polymers and natural biomaterials have been widely utilized^6, 16–19^. Although some success has been reported in resolving mechanical and biological concerns, appropriate microstructure is another critical point which needs to be addressed to improve the outcome of tissue regeneration^20^. The microstructure is essential for promoting the formation of intima and media as the two major cell layers of native vascular wall^15^. Smooth muscle cells (SMCs) with a contractile phenotype are the main cell type in the vascular media while lumen is coated with endothelial cells (ECs)^21^. Therefore, the best hemocompatibility is obtained when the luminal surface is coated with healthy endothelial cells that facilitate blood flow and prevent thrombosis^22, 23^, while the presence of functional SMC within the medial layer of vascular wall facilitates certain functions such as vasoconstriction and relaxation as well as secretion of vasoactive regulators^21, 24^. Smaller pores are more suitable for ECs to create a monolayer on the luminal surface of the scaffold while SMCs require interconnected pores above 60 micrometers in diameter to proliferate and build a muscular media^24, 25^. Therefore, an ideal vascular scaffold must provide the suitable requirements for the growth of both cell types.

Silk fibroin (SF) is an advanced naturally-derived biopolymer that offers several features including unique mechanical properties, versatile processability to different formats, proper biocompatibility, very low immunogenicity and adaptable biodegradability,which make it a perfect choice for vascular scaffolds^26, 27^. Also, it has been revealed that the SF is more biocompatible than other commonly used polymeric degradable biomaterials such as PLA, PGA and collagen^28^. According to the above-mentioned parameters, SF is a promising option among the polymeric materials for scaffolding in the regeneration of vascular grafts. Hence, various methods have been employed for the fabrication of small-diameter silk-based vascular grafts such as electrospinning, gel spinning, dipping and bilayered grafts composed of SF or composites of SF with other polymers which have been appraised in *in-vitro* and *in-vivo* settings^6, 29–32^.

Complication of foreign material at the blood-material interface is a critical issue in vascular tissue engineering which is the subject of many studies, but the challenges still remain. To overcome this issue, the luminal surface of the vascular graft should hold anticoagulant activity as long as the endothelial layer achieves full development which would prevent the early failure and improve the chance of successful vascular regeneration^23, 33, 34^. To this end, heparin, an anticoagulant drug, has been used to improve antithrombogenicity of the vascular grafts using various methods^23, 35, 36^. Although there have been lots of improvements in vascular graft constructions over the past decades, further studies are still needed to be performed to improve the applicability of these small-diameter vascular grafts.

In this study, a composite tubular scaffold was made by embedding braided silk fibers into lyophilized SF sponge followed by covalently coating heparin on the intimal layer via layer-by-layer self-assembly of heparin and HIC. This new approach resulted in desirable mechanical and microstructural characteristics. The as prepared scaffold was analyzed by comprehensive morphological, structural and mechanical characterizations. The mechanical properties of the tubular scaffold were characterized according to the standard ISO 7198 “Cardiovascular implants – Tubular vascular prostheses”^17^ and compared with those of other vascular grafts and native human saphenous veins. Hemocompatibility of the scaffolds was assessed by analyzing the stability of heparin coating, *in vitro* antithrombogenic properties and hemolysis. In addition, calcification of the scaffolds was investigated by an ectopic implantation in Sprague Dawley rats over a period of 12 weeks. Finally, human umbilical vein endothelial cells (HUVECs) were used to evaluate the cytocompatibility of the scaffolds. The results of this study suggest that the prepared scaffold in this way can be a promising candidate for engineering-based regeneration of vascular tissues.

## Results and Discussion

### Fabrication of vascular scaffold

Various techniques have been developed to prepare silk-based vascular grafts in previous studies such as electrospinning, gel spinning, dipping as well as bilayer structures, including sponge coating and freeze-drying, that met vascular graft requirements in some aspects^5, 6, 9, 29–32, 37–39^. Although these grafts showed remarkable features such as good biocompatibility and outranged in some mechanical properties, there are still other critical properties needed to be improved including blood compatibility, flexibility without kinking and appropriate microenvironment for SMCs growth^9, 15, 33^. In the present study, a relatively simple method without numerous controlling parameters was utilized to prepare a composite tubular scaffold by the combination of braided and freeze-dried SF in order to overcome the mentioned issues. Incorporation of freeze-dried SF into braided silk fibers creates a mechanically favorable structure with a controlled microstructure that is essential for cells growth and tissue remodeling.

Moreover, in this investigation, in order to improve the blood compatibility of the prepared vascular graft, the inner wall was modified with heparin as an anticoagulant agent. To ensure the durability of the antithrombogenic property of the vascular graft before confluent coverage of ECs, 6 cycles of heparin coating were applied to form the desired thickness. After 6 cycles of loading, 1.48 ± 0.19 mg/cm^2^ heparin was attached, i.e. accumulated from loading of 246 ± 32 μg/cm^2^ per cycle. The binding of heparin to the surface of the intimal layer not only provides the maximum efficiency for loaded heparin due to direct exposure of blood to the drug but also might hinder its inhibitory effect on proliferation of SMCs in media. In addition, this method possibly provides the feasibility of incorporation of growth factors like VEGF at the exact site of ECs growth even after utilization of organic solvents or alcohol treatment.

### H-CSVS structure

**Figure 1:**
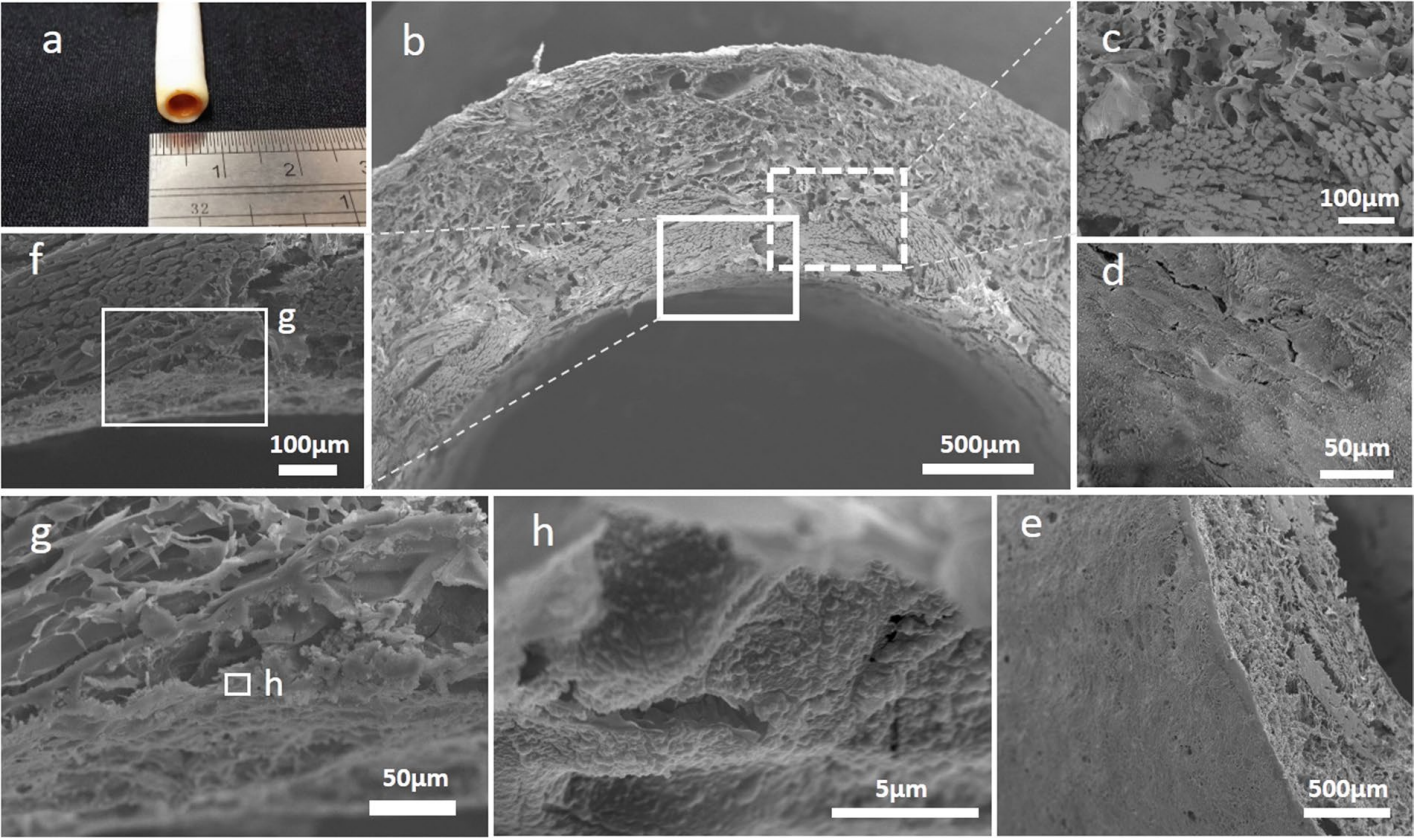
Representative morphological assessment of the scaffold. (**a**) Macroscopic appearance of the 4mm diameter H-CSVS. (**b**) SEM image of cross-sectional portion of the H-CSVS. (**c**) SEM image of interface between mesh tube and outer layer scaffolds. (**d**) SEM image of the luminal surface of the tubular scaffolds. (**e**) SEM image of outer surface. (**f**) Interface between mesh tube and luminal surface and (**g**,**h**) the coated heparin on the intimal layer of the vascular scaffold.

It has been reported that freezing temperature and consequently freezing speed are the most influential factors in tailoring the morphology and pore size of freeze-dried scaffolds. By decreasing the freezing temperature, more crystal cores will form while they have less time and space to grow, and after the ice crystals removal, the smaller pores will remain^40^. Therefore, the lower temperature (−80 °C) was utilized to form the internal layer of scaffold while the external portion was frozen at −20 °C and then the morphology was examined by scanning electron microscopy (SEM) (Fig. 1). Moreover, the second lyophilization step during the creation of the outer layer of the scaffold may contribute to further reducing the pore size of luminal layer and formation of a thin, smooth and continuous layer of SF between the internal cylindrical mold and the mesh tube (Fig. 1d). This surface was then coated with a sub-micrometric layer of heparin and HICs (Fig. 1h). Heparin-HICs, as observed by orange color, only present on the inner layer of the tube and is separated from the outer layer by a distinct visual boundary (Fig. 1a). It was demonstrated that smaller pores in scaffolds could improve ECs proliferation^25^. Conversely, larger pores (60–150 μm) in the outer layer could aid SMCs cultivation, infiltration and proliferation^24^. So, unlike electrospinning^5^ and gel spinning techniques^30^, fabrication of silk scaffold through the mentioned method provides a layered structure with different features in the same context, to carry the desired aspects for the growth of both main cell types in vascular tissue regeneration^15^.

Using a low concentrated silk aqueous solution (2% w/v) with relatively higher freezing temperature (−20 °C), a highly interconnected porous media with a pore size of above 60 μm was generated which is suitable for SMCs in-growth (Fig. 1b,c)^24, 41^. There was no sign of delamination in the fabricated structure while the mesh was completely embedded in the matrix (Fig. 1b,c,f). The silk fibers were dismembered into the naturally synthesized filaments with a diameter of about 10 μm (Fig. 1c). The thickness of the inner mesh layer was about 350 μm, while that of the outer layer was about 700 μm (Fig. 1b). The thickness of the outer layer can be altered by adjusting the space between the two modules. It’s also observed that during the freeze-drying process, a skinny and compact layer with low porosity would be generated at the interface of air and frozen solution which could be beneficial in vacuum cell seeding and prevention of cells to be washed out by blood pressure (Fig. 1e)^42^.

**Figure 2:**
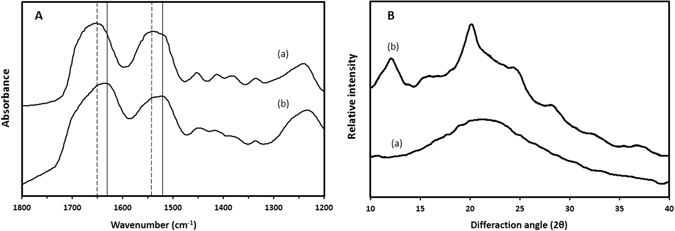
(**A**) FTIR spectra and (**B**). XRD pattern of scaffolds prepared from SF aqueous solution, (a) before and (b) after treatment with 70% methanol for 30 min.

The changes in the molecular structure of silk scaffold were studied by Fourier transform infrared spectroscopy (FTIR) (Fig. 2A). The scaffold formed immediately after lyophilization demonstrates random structure (1651 cm^−1^ amide I, 1541 cm^−1^ amide II and 1236 cm^−1^ amide III)^32, 34^ which was transformed to β-sheet conformation (1627 cm^−1^ amide I and 1519 cm^−1^ amide II) after treatment with 70% methanol for 30 min. Also, the peaks at 1232 cm^−1^ and shoulders at 1654 cm^−1^ and 1541 cm^−1^ indicated the existence of some random/α-helix conformation in the samples^34, 43^.

XRD experiments were also performed to confirm the structural changes of SF (Fig. 2B). The broad peak appeared before methanol treatment demonstrated the amorphous state of silk in the scaffold^32^. After soaking in methanol, the silk scaffolds showed some distinct diffraction peaks appeared at 2θ values of 12.2° (I), 20.2° (II), 24.3° (I) and 28.2° (I), indicating the formation of crystal structure (silk I and silk II) of SF^43^. The conformation change to silk I and silk II structure is essential for the creation of water insoluble silk-based scaffolds and subsequently maintenance of its mechanical properties and integrity in aqueous environment^44^.

### Mechanical properties of the H-CSVS

A biomimetic tissue engineered vascular graft should not exhibit mechanical mismatch in comparison to the native artery when imposed to physiological range of stimulation, while it may even show different behavior in other tensions. An ideal vascular graft should have acceptable suture retention strength to ensure that the graft would not detach at the anastomose site and also should have sufficient strength to tolerate arterial pressures and pulsatile blood flow to hinder aneurism. In addition, a functional vascular graft should be such elastic that it can bend to sufficient radius for clinical usage without kinking and also show similar compliance to native arteries to eliminate intimal hyperplasia and restenosis^12, 45^.

It is noteworthy that the regenerated silk fibroin is a stiff material that raises some limitations in the design of vascular grafts^46^, meaning that scaffolds with larger pores reveal have unsuitable strength and those with smaller pores suffer from low flexibility which results in kinking in required bending radius^32, 37^. In addition, owing to this stiffness, the higher compliance could be achieved by diminishing the wall thickness and mechanical strength as a result^5^. The novel scaffold in the current study represented anti-kinking radius of about 2 cm, indicating admirable flexibility for clinical usage. Furthermore, the scaffold also offered higher suture retention strength and burst pressure than previously fabricated silk tubes^5, 32, 47^. However, it displayed comparable elastic modulus with human native artery (1 MPa)^48^. For a comprehensive comparison, all the measured mechanical properties of H-CSVS and other vascular substitutes were summarized in Table 1.

**Table 1:** Summary of the mechanical properties of some vascular grafts and native tissues (means ± standard deviations).

Sample	Dynamic compliance (% per 100 mmHg)	Burst Pressure (mmHg)	Suture retention strength (N)	Elastic modulus (MPa)
This work	2.46 ± 0.56	>10000 mmHg	6.1 ± 1.7	1.54 ± 0.44
Electrospun SF^5^	3.51 ± 0.42	575 ± 17	—	—
Bilayered SF	2.3 ± 0.5	806 ± 185	5.6 ± 0.9	17.9 ± 3.2
PTFE^45^	0.2–0.9	2580–8270	2.45–11.77	918 ± 52.9^30^
Saphenous vein^45^	0.7–2.6	1600–2500	1.76–2.45	—

The mentioned mechanical properties are prerequisite conditions for a vascular graft to be successfully implanted and allow the blood to pass through. However, for the long-term efficiency, the graft should have a compliance matching that of host tissue to prevent intimal hyperplasia and restenosis. The measured value of dynamic compliance for H-CSVS was 2.46 ± 0.6 (×10^−4^ mmHg) matched with that of saphenous veins (0.7–2.6% per 100 mmHg), which would prevent problems associated with a compliance mismatch^49^. The enhanced compliance value compared to previously fabricated silk tubes^16, 29, 39^ is due to the braided structure of silk yarns cooped in a highly porous silk scaffold. This specific conformation provides the ability of angular movement for silk fibers in a way that the diameter and braiding angle are dependent on each other. While the biomechanical properties of the commercial grafts did not match all those of a human vein, the present construct simultaneously provided all the biomechanical properties (flexibility, suture retention strength, burst pressure, and compliance) in the acceptable range compared to previous silk-based vascular grafts with adequate values for only some of biomechanical criteria^5, 29, 39^. These results were obtained without eliminating radial interconnectivity^6, 32^ or decreasing the wall thickness which leads to burst pressure decline and lack of proper layer for formation of muscular media^5, 47^, thus providing an improved alternative for small-diameter vascular grafts.

Although H-CSVS exhibited a similar compliance to saphenous vein, this result is partially limited by the assumption of the porous SF scaffolds as incompressible material. The assumption of incompressibility, although is absolutely valid for the native blood vessels tests, might have introduced an underestimation in the compliance measurements. This assumption was used to maintain a consistent protocol while comparing SF scaffold with native vessels^12^.

### Release characteristic of heparin from modified scaffolds

**Figure 3:**
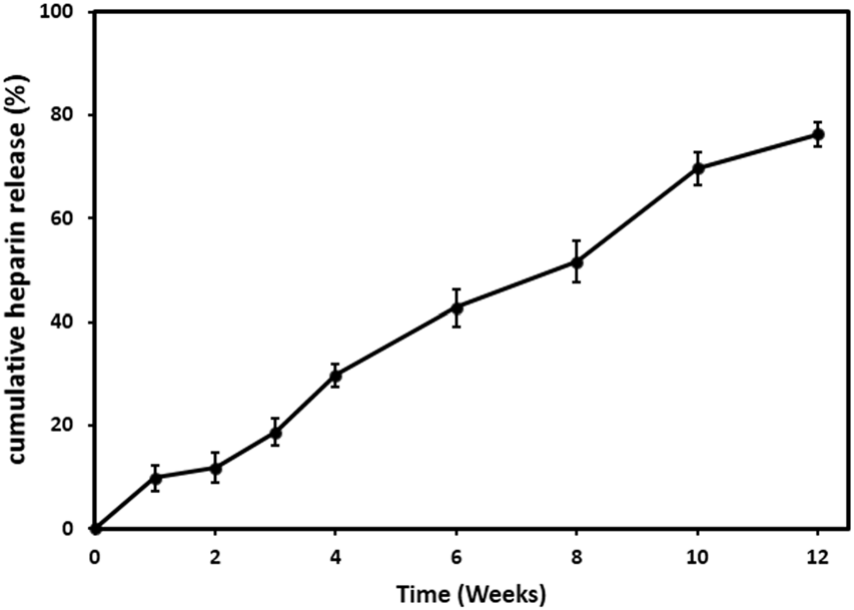
Cumulative release profile of heparin from H-CSVSs over 12 weeks.

The biocompatibility of SF is widely acknowledged, but the issue related to foreign material in contact with the blood still remained unresolved^33^. To overcome this challenge, heparin, a widely used anticoagulant biopolymer, has been used in numerous vascular engineering applications^8, 23, 35, 36, 50^. To achieve the desired outcome, the heparin must exist at the blood-material interface in an adequate dose for a sufficient period. The most noticeable disadvantage of common blending methods is the unsteady release characteristic consisting a burst release which a large percentage of drug is released from the material in a short period of time^32, 50^. It means that after a few days, the rate at which the drug is released becomes less than effective. Herein, our efforts were made to overcome this challenge by the use of multi-layer chemical absorption of heparin. Figure 3 represents the percentage of the heparin released from the surface of H-CSVSs in PBS solution over a period of 12 weeks. The scaffolds preserved more than 23% of their primary heparin content at the end of the washing period. Heparin release was accomplished by a rather constant rate and no burst release occurred during the experiment. Such behavior can be explained by covalent linkage between heparin molecules and iron ions that must be hydrolyzed to release heparin into the solution. Since the heparin and HIC assembled layer-by-layer on each other and all the bonds are the same in the entire layers, the release rate is approximately constant and controlled by bond hydrolysis.

This method prolonged the release duration of heparin from SF at least 3 times higher than the maximum value reported for silk scaffolds^32^, thus indicating its ability to induce antithrombogenicity to silk-based vascular grafts.

### Blood compatibility

One of the most important features leading to the privilege of vascular graft is its antithrombogenic activity, which should obtain long-term prevention of clot formation *in vivo*. Recently, APTT and PT tests, which are widely used in clinical detection of the abnormality of blood plasma and primary screening of anticoagulating chemicals, have been applied to evaluate *in vitro* antithrombogenicity of biomaterials^33^. The blood coagulation cascade includes intrinsic, extrinsic, and common pathways. APTT test indicates the duration in which the intrinsic pathway is activated while PT represents the extrinsic and common blood coagulation activation^3^.

Prothrombin time (PT) and activated partial thromboplastin time (APTT) of samples were measured in order to study how the antithrombogenic activity of scaffolds preserved after washing for 12 weeks (Table 2). The PT and APTT of the unmodified scaffolds were considered to be in the normal reference range (13.6 ± 1.1 s for APTT and 26.5 ± 2.4 s for PT). For heparinized scaffolds, these values exceeded the upper range of the clot detection instrument (>180 s for APTT and >120 s for PT) even after 10 weeks of washing. This significant improvement in antithrombogenicity compared to other heparin modification methods^32, 50^ is because of strong covalent linkage of heparin on the surface which is hard to be removed and, in the meantime, the surface that blood encounters is still heparinized regardless of coating thickness. At week 12, coagulation time was decreased to the detection range of clot detection instrument (151.5 ± 11.7 s for APTT and 85.6 ± 9.1 s for PT). Considering the quite constant slope of heparin release profile, these decrease is possibly due to structural changes of heparin molecules.

**Table 2:** Prothrombin time (PT) and activated partial thromboplastin time (APTT) of heparinized and non-heparinized scaffolds over 12 weeks.

Time (weeks)	heparinized								Non-heparinized SF scaffold
	1	2	3	4	6	8	10	12	
PT	>120	>120	>120	>120	>120	>120	>120	85.6 ± 9.1	13.6 ± 1.1
APTT	>180	>180	>180	>180	>180	>180	>180	151.5 ± 11.7	26.5 ± 2.4

There was a significant difference between clotting times for two types of scaffolds at each point of time (P < 0.01).

This significant time independent difference in antithrombogenic activity between the unmodified and heparin modified scaffold groups (P < 0.01) demonstrated the advantage of this coating method as an improved approach for small-diameter silk-based vascular grafts.

**Figure 4:**
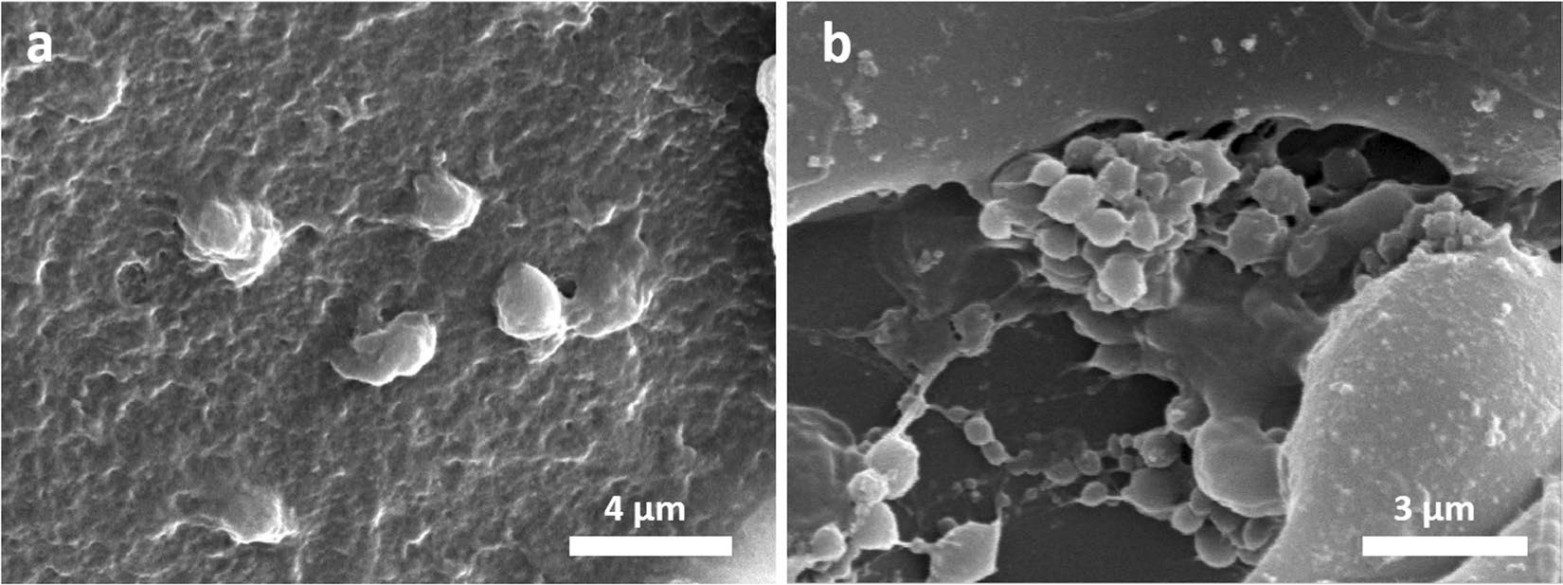
SEM images of adherent platelets on the surface of heparinized (**a**) and non-heparinized SF scaffolds.

To study platelet adhesion after heparin loading, SEM images were analyzed to confirm the antithrombogenic activity of scaffolds (Fig. 4). Relatively large numbers of activated platelets were adhered to and formed clusters on the surface of unmodified scaffolds (Fig. 4b) while few platelets were observed as discrete spherical entities on the modified scaffolds (Fig. 4a).

Hemolysis is another issue associated with the blood compatibility of biomaterials that should be analyzed for circulating blood contacting devices, according to ISO 10993.4:2009 standard^51^. The hemolysis rates were 1.1 ± 0.3% and 1.9 ± 0.2% for heparinized and non-heparinized scaffolds, respectively. Although heparinization of silk scaffolds significantly decreased the hemolytic activity (P < 0.05), both samples are classified as non-hemolytic materials^3^, demonstrating excellent blood compatibility of this vascular scaffold.

### Calcification behavior

**Figure 5:**
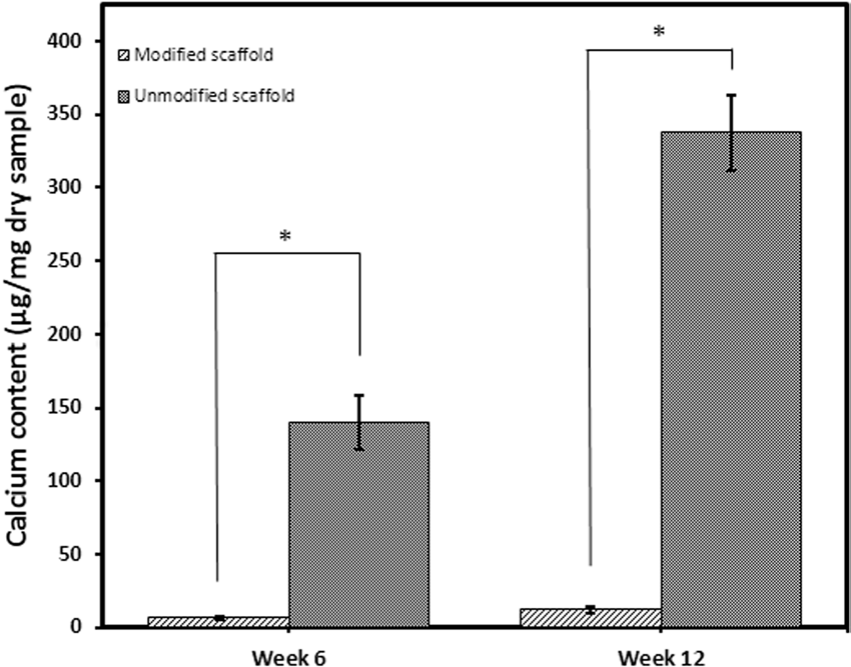
Column graph of calcification behavior of heparinized and non-heparinized silk scaffolds *in vivo*. There was significant difference between calcium content in two types of scaffolds at each time point (P < 0.01).

Generally, calcium deposition in vascular grafts is a reason for thrombotic occlusion and graft failure in the long term. Deposited calcium could reduce the permeability by cramping the pores up and may result in stiffening and subsequently tapering the compliance over time^52^. Thus, it should be considered as an important factor in the design of vascular grafts to express calcification resistance as much as possible. It has been reported that heparin and iron ions can protect biomaterials from calcification^36^. The measured calcium contents for modified and unmodified scaffolds at week 6 were 6.69 ± 0.96 and 139.93 ± 18.82 μg/mg of dry weight, respectively. And at week 12, the amounts obtained for modified and unmodified scaffolds were 11.97 ± 2.48 and 337.53 ± 25.99 μg/mg dry weight, respectively (Fig. 5), being in agreement to previously reported results^36^. There was a significant difference in calcium content between the heparinized and non-heparinized scaffolds at both time points (P < 0.01), indicating the advantage of H-CSVSs for *in vivo* vascular regeneration.

### Histological analysis

**Figure 6:**
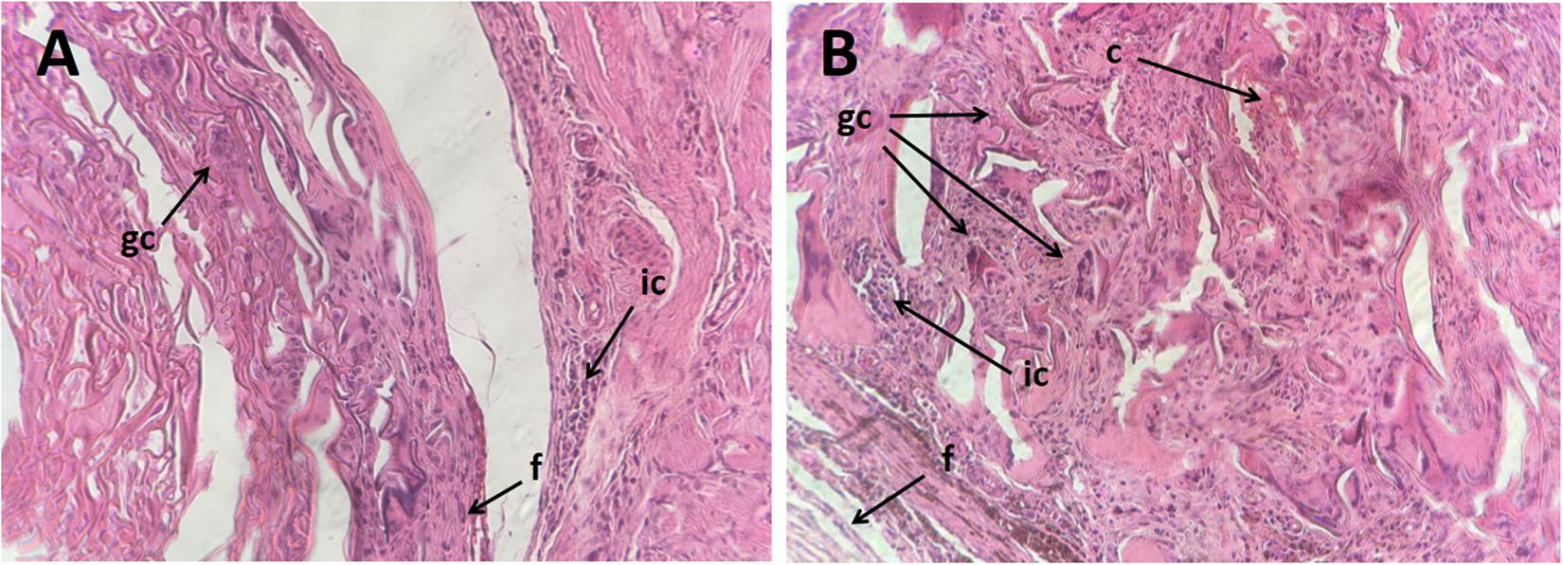
Representative images (20×) of H&E stained slides of subcutaneously implanted samples in rats, 6 weeks post-surgery showing tissue sites around (**A**) heparinized and (**B**) non-heparinized scaffolds. Abbreviations used: ic = inflammatory cells, gc = giant cells, f = fibrous connective tissue, c = capillary.

Histolopathological evaluation of H&E stained tissue sections exhibited variable distributions of different inflammatory cell densities for heparinized and non-heparinized scaffolds. As shown in Fig. 6, the granulomatous infiltration can be seen in both groups. On the other hand, a minor formation of fibrous connective tissue was noticed as well as a mild infiltration of mononuclear inflammatory cells which had more even distribution in heparinized scaffolds. There were occasional multinucleated giant cells observed in non-heparinized scaffolds while it was more in the heparinized scaffolds. Also, some capillaries were found in heparinized scaffolds which is in accordance with previously published results^20^.

### Cell compatibility

Endothelial cells are the most vital cell type in vascular tissues which, therefore, their presence on luminal surface is mandatory to sustain a small-diameter vascular graft patent^22, 23^. Therefore, in the present study, we chose HUVECs to analyze biocompatibility of the grafts.

**Figure 7:**
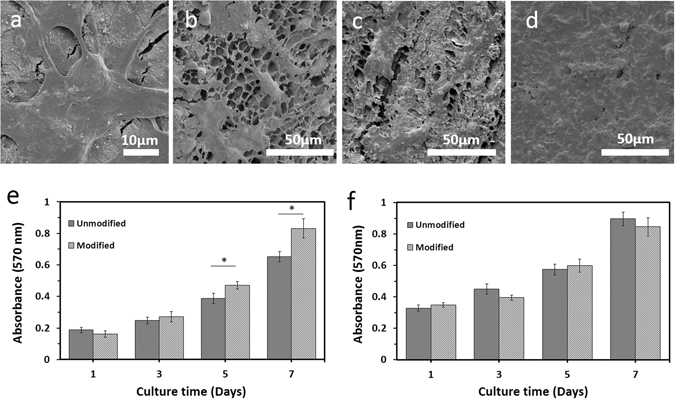
Biological characterization of composite SF vascular scaffolds. (**a**) SEM image of flattened morphology of HUVECs on luminal surface of H-CSVS. (**b**–**d**) SEM image of HUVECs cultured for 1, 3 and 7 days on lumen of H-CSVSs, respectively. (**e**) MTT cell viability assay with HUVECs showing good cytocompatibility of the H-CSVSs. The viability of HUVECs increased linearly with culture time. Similar results were found for control (unmodified SF scaffold). (**f**) MTT cell viability assay with A7r5 cells showing no significant difference between smooth muscle cell growth on external layer of H-CSVSs and controls. Asterisk (*) indicates significant difference (P < 0.02).

Analysis of SEM images showed the ECs being uniformly adhered and proliferated on the lumen of H-CSVSs with spread and flattened morphology (Fig. 7a). ECs successfully adhered to the surface within 24 h and as cultivation goes on, the coverage was expanded and a cellular monolayer was formed by day 7 (Fig. 7b–d).

**Figure 8:**
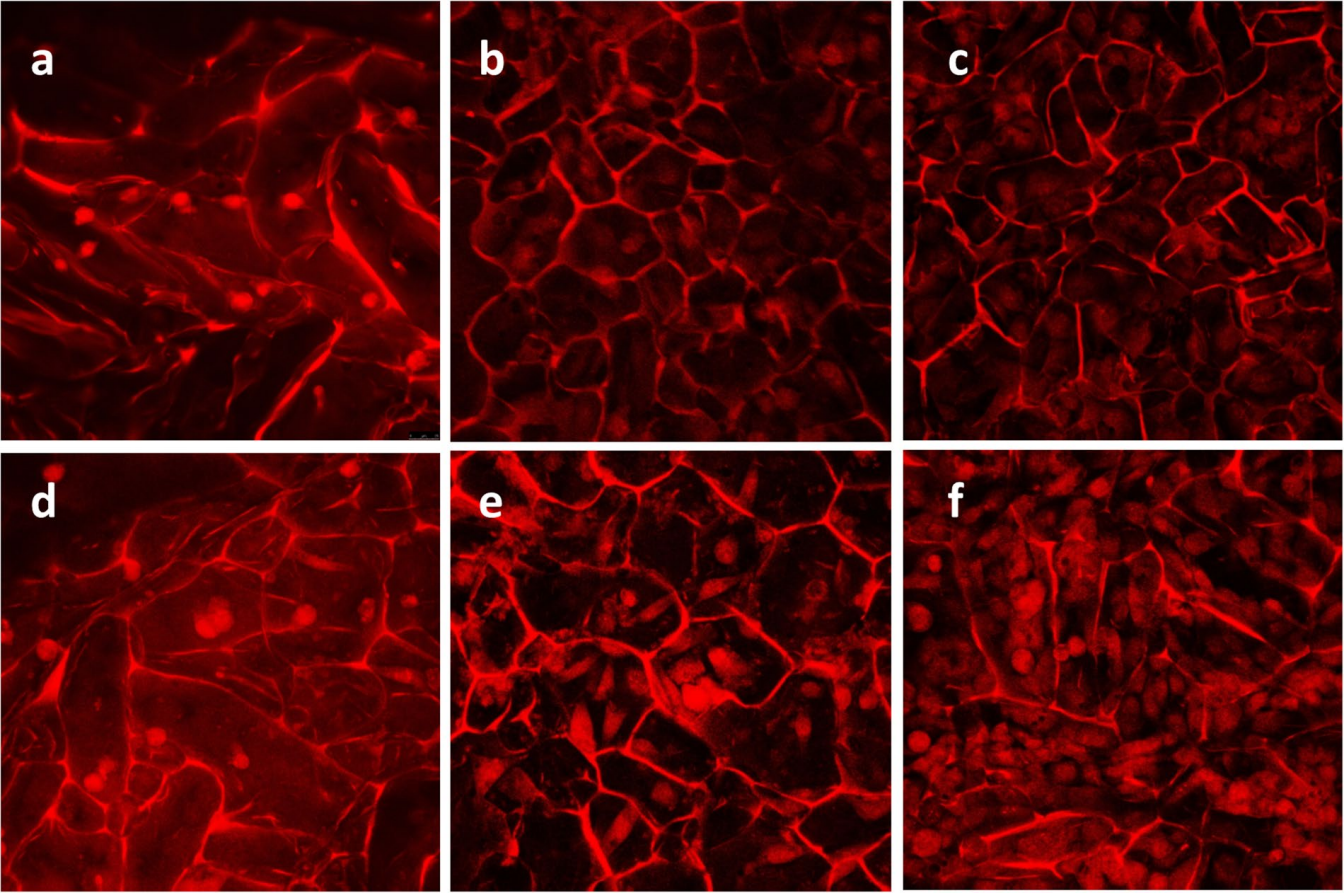
Growth and proliferation of human umbilical vein endothelial cells on the scaffolds were studied using a confocal microscope. Human umbilical vein endothelial cells were assessed after 1, 3, and 6 days of cultivation by PI staining on heparinized (**d**–**f**) and non-heparinized scaffolds (**a**–**c**). Images (30×) were captured from the surface to a depth of 85 μm with excitation/emission at 532/615 nm.

The proliferation and presence of HUVECs on the surface of scaffolds were studied by confocal microscopy and revealed the increase of cell population after cultivation (Fig. 8). Both heparinized (Fig. 8d–f) and non-heparinized (Fig. 8a–c) scaffolds effectively supported cell attachment and proliferation.

These results were confirmed by tracking the metabolic activity of ECs on scaffolds. As shown in Fig. 7e, the viability of cells increased linearly on both scaffolds, indicating the ability of scaffolds to support the proliferation of ECs without producing any toxic effects for at least 7 days. Comparing the cell viabilities cultured on heparinized and non-heparinized scaffolds, there were no significant differences between the first and third day. However, the cell viabilities cultured on modified scaffolds increased significantly after 5 days in culture (P < 0.02), which is consistent with reported effects of heparin on the growth of endothelial cells^53^.

MTT assay of A7r5 cells cultured on the outer sponge of scaffolds which their inner layers were loaded with heparin did not differ from the sponge of scaffolds without heparin loading (Fig. 7f), demonstrating the suitableness of the heparin loading method in the current study regarding emitting no adverse effect on growth of vascular muscle cells. Cell culture *in vitro* indicated that the H-CSVS might be a suitable scaffold for small-diameter blood vessel reconstruction.

## Methods

### Preparation of aqueous silk fibroin solution

Cocoons of Bombyxmori silkworm were kindly supplied by Dr. Hosseinimoghadam (University of Guilan). Silk fibroin solution was prepared as previously described^43^. Briefly, the B.mori cocoons were firstly cut into small pieces and boiled in 0.02 M Na_2_CO_3_ (Merck, Germany) aqueous solution for 20 min to remove the glue-like sericin protein, and then rinsed thoroughly with deionized (DI) water to remove the residual sericin. The extracted SF was then dissolved in 9.3 M LiBr (Merck) solution at 60 °C for 4 hours yielding a 20% (w/v) solution. This solution was dialyzed against DI water using dialysis tubing cellulose membrane (MWCO = 3.5 kDa, Sigma-Aldrich) for 72 hours to remove the salt. The final concentration of SF aqueous solution was approximately 7% (w/v), which was determined by weighing the solid mass of a known volume of solution after drying.

### Preparation of composite vascular graft

**Figure 9:**
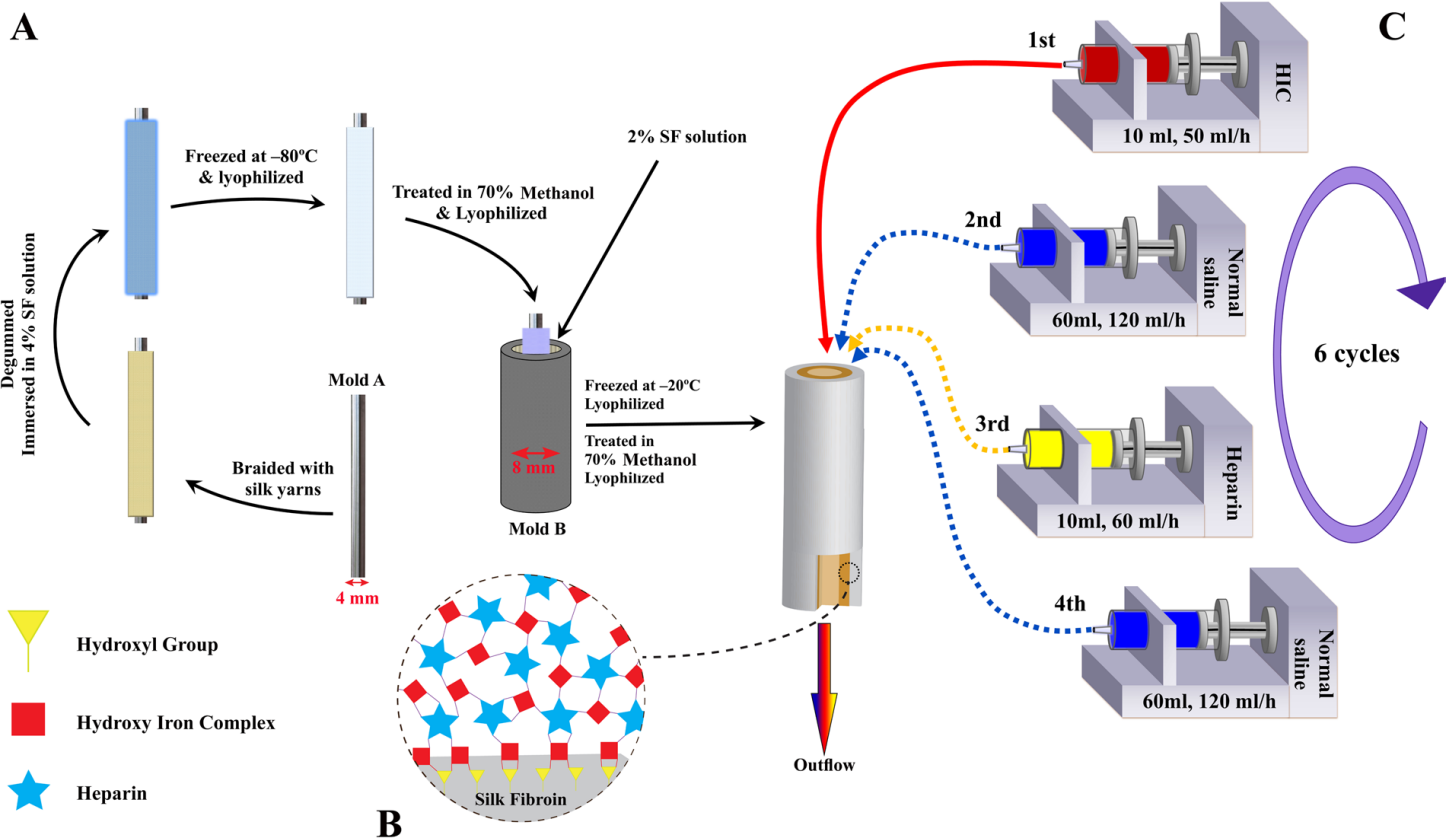
(**A**) Scheme of composite silk-based vascular scaffold (CSVS) fabrication, (**B**) illustration of multilayered coating of heparin and HIC on SF surface, (**C**) process of heparin loading on the lumen of the scaffold.

Considering that SF is a tough biomaterial and has a very high modulus, utilization of homogeneous structure with low porosity or casted films in the construction of vascular grafts may result in limited flexibility and brittleness in the dry state in some cases. On the other hand, the usage of structures with high porosity in vascular grafts fabrication could cause mechanical failure and blood leakage^17, 38^. Therefore, degummed silk fibers were used to tailor the porosity and pore size distribution without affecting the mechanical properties. The tubular mesh was produced by twisting the silk yarns (each yarn consists of twelve 21-denier fibers) around a stainless steel mold (4 mm in diameter) (Fig. 9, mold A) with a high-speed rope braiding machine (JC 2–24, Zhangjiagang Textile Machinery Co., People’s Republic of China) under controlled process conditions. To be more precise, the yarns were twisted around a cylindrical mold with a 2/2 pattern at an adjusted take-up rate to have the mesh tubes with a braid angle of ~50°. The mesh tubes were boiled in an aqueous solution of 0.02 M Na_2_CO_3_ for 30 min during which the tubes were removed every 10 minutes and ultrasonicated in the same solution at 80 °C for 2 min and then returned to a fresh boiling solution. The meshes were then rinsed thoroughly with deionized water to remove the possibly remained sericin proteins and other impurities. The dried tubes were soaked in 4% (w/v) SF aqueous solution and subsequently frozen at −80 °C for 12 h followed by lyophilizing for about 48 h. In the next step, to induce crystallization and insolubility in water, scaffolds were treated with 70% methanol for 30 min followed by washing with deionized water and further lyophilization. The prepared structure was then put in the middle of a cylindrical mold (Fig. 9, mold B) and subsequently filled with 2% (w/v) SF aqueous solution and frozen at −20 °C for 12 h. Lyophilization was carried out for about 72 h to create outer porous layer of the tubular wall. Finally, the CSVS were treated with 70% methanol for 30 min to induce crystallization and insolubility in water (Fig. 9A).

### Loading of heparin

HIC was used as a linker between heparin (Heparin Sodium, Molecular weight 10,000–15,000, 204 IU/mg, Hepalink, China) and silk proteins in order to create a heparinized CSVS (H-CSVS) (Fig. 9B). HIC solution was prepared as described previously^36^. Briefly, FeCl_3_⋅ 6H_2_O was dissolved in deionized water and its pH was slowly adjusted to 3.0 with NaOH solution while stirring. The final concentration of Iron ions was adjusted to 0.05 mol/L by deionized water. The modification procedure was as follows: 10 ml of HIC solution was placed in a 10 ml syringe and the tubular scaffold was connected to the syringe through a hose. The solution was pumped through the lumen of the scaffold by the syringe pump with a flow rate of 50 ml/h followed by passing 60 ml normal saline through tubular scaffold by a flow rate of 120 ml/h to rinse the non-adhered HIC. Then, 10 ml of 5 mg/ml heparin solution containing 0.9% NaCl was passed through the scaffold’s lumen for 10 min at the room temperature. Finally, 60 ml normal saline was passed through tubular scaffold by a flow rate of 120 ml/h to rinse the non-adhered heparin (Fig. 9C). This cycle was repeated 6 times and then the scaffolds were rinsed with normal saline and lyophilized for about 48 h and stored at 4 °C for future use.

### Quantification of linked heparin

The elemental analysis equipment SPECTRO ARCOS inductively coupled plasma optical emission spectroscopy (ICP–OES, SPECTRO Analytical Instruments, Kleve, Germany) was used to measure the amount of linked heparin. To this end, the calibration curve of heparin concentration versus the amount of sulfur present in heparin molecules was plotted. Since the concentration of sulfur is directly proportional to the concentration of heparin in the solution, its concentration was measured before and after passing the heparin solution through the tubular scaffold in the modification process. The amount of linked heparin per surface area was calculated as follows:

1 $${{\rm{L}}}_{{\rm{H}}}=\frac{{H}_{i}{V}_{H}-{H}_{o}({V}_{H}+{V}_{s})}{{A}_{l}}$$

where L_H_ is the linked heparin per surface area in each assembly cycle, H_i_ is the initial concentration of heparin, H_o_ is the concentration of heparin in collected solutions of heparin and salt which was passed through the tube, A_l_ is the luminal surface area and V_H_ and V_S_ are the volume of passed heparin and salt solution, respectively. The total amount of linked heparin per surface area (L_T_) was calculated using the following equation:

2 $${{\rm{L}}}_{{\rm{T}}}={\sum }_{1}^{6}{{\rm{L}}}_{H}$$

### Structural analysis

Scanning electron microscopy (SEM; Hitachi SU3500, Tokyo, Japan) was used for morphological evolution of the H-CSVS. The cross section of the freeze-dried scaffold was cut by a sharp razor blade. The specimens were mounted on aluminum stubs using double adhesive conductive carbon tape and sputter coated with gold to a thickness of 10 nm prior to imaging.

The structural change of the scaffolds material was analyzed by a Fourier Transform Infrared (FTIR) spectrometer (ABB Bomem, MB-100). For each measurement, 32 scans were coded with a resolution of 4 cm^−1^, with the wave number ranging from 400 to 4000 cm^−1^.

To investigate the crystallinity of the samples, XRD analysis was also performed with an X-ray diffractometer (XRD, PANalytical, X’Pert PRO MPD, The Netherlands) with CuKa radiation at 40KV and 40 mA and scanning rate of 0.01 s^−1^. The dried samples were pressed into sheets with a hydraulic compressor prior to the examination.

### Mechanical properties

#### Dynamic compliance

Dynamic compliance was measured by a custom designed vascular perfusion system according to previously described system^12^. Three vascular scaffolds of 3 cm length were used to measure the dynamic compliance under cyclic pressure. In order to reduce the permeability of the tubular scaffolds, a highly deformable balloon was introduced to samples, in accordance with the standard practice ISO 7198^5^. Specimens were secured at either end to a nipple with a Teflon tape and placed in a chamber of phosphate buffer saline (PBS) maintained at 37 °C. The chamber was filled with PBS which circulated continuously around the tube to maintain a temperature of 37 °C and a pressure of 0 mmHg. Simultaneously, PBS was pumped through the lumen of the vascular scaffold using a biomedical centrifugal pump to deliver physiological arterial pulsatile pressure (i.e. 1 Hz, 80/120 mmHg systolic/diastolic) and flow (120 ml/min). A pressure transducer (WIKA’ Germany) capable of measuring dynamic pressure up to 200 ± 2 mmHg was placed upstream of the tubes to measure intraluminal pressure. The external diameter of the pressurized tubes was measured with a custom-made optic micrometer with an accuracy of 2 micrometers. Prior to insertion into the system, the silk tubes were soaked in PBS at 37 °C for 24 hours. Both pressure and diameter signals were automatically recorded at 120 Hz for 1 min every hour over 8 h using an acquisition data instrument (Advantech, Taiwan) connected to a personal computer.

Compliance of system (C_sys_) was calculated from recordings of pressure (P) and the inner diameter (ID_p_) as:

3 $${{\rm{C}}}_{{\rm{sys}}}=\frac{1}{{P}_{120}-{P}_{80}}\frac{I{D}_{120}-I{D}_{80}}{I{D}_{80}}$$

where P_120_ = 120 mmHg and P_80_ = 80 mmHg. ID_p_ was estimated on the assumption of incompressibility from the cross-sectional area (A) and the outer diameter (OD_p_) of the tubes (measured with an optic micrometer) using the following equation:

4 $${{\rm{ID}}}_{{\rm{p}}}=\sqrt{{(O{D}_{p})}^{2}-(4A/{\rm{\pi }})}$$

The compliance of SF tubes was calculated using the ensuing compliance formula^6^:

5 $$\frac{1}{{C}_{{\rm{S}}{\rm{y}}{\rm{S}}}}=(\frac{1}{{C}_{balloon}}+\frac{1}{{C}_{SF}})$$

6 $${C}_{SF}=(\frac{{C}_{sys}{C}_{balloon}}{{C}_{balloon}-{C}_{sys}})$$

#### Burst pressure

Burst pressure was measured by increasing the internal pressure of the 3 cm long tubular scaffolds with vaseline, while submerged in a saline bath maintained at 37 °C via a heat exchanger to keep hydration and to simulate *in vivo* conditions. Vaseline was infused at 0.1 ml/min with a 20 ml syringe and the pressure was recorded via a high-pressure gauge (WIKA, Germany) after clamping the downstream line. The maximum sustained pressure before scaffold rupture was taken as the burst pressure^17^.

#### Longitudinal property

The longitudinal mechanical property was determined using a uniaxial tensile testing machine with a 500 N load cell (Hounsfield H10KS, UK). Samples (L = 3 cm) were first soaked in PBS at 37 °C for 2 hours and then fixed in the clamps of the testing device with a 10 mm inter-clamp distance. Specimens were pulled at a crosshead speed of 5 mm/s with a preload of 0.1 N while hydrated every minute. The Young’s modulus was calculated by measuring the slope of the elastic region in the stress–strain curve^32^.

#### Suture retention strength

Suture retention strength of the H-CSVSs was also measured with the uniaxial tensile testing machine. One end of the tubular specimen (L = 2 cm) was fixed with the stage clamp of the tester and a 4-0 monofilament suture (SUPA Medical Devises, Iran) was passed through the wall of another end of the graft, 2 mm from the edge. The suture was connected to another clamp and pulled at a rate of 8 mm/min until pull-out. The maximum force recorded before pull-through of the suture considered as suture retention strength. This test was repeated three times at 120° intervals. Prior to analysis, samples were soaked in PBS at 37 °C for 2 hours and tested in wet condition^32^.

### Release of heparin from scaffold surface

To investigate the stability of coated heparin on the scaffold surface, the shaken wash model was used to simulate the *in vivo* conditions of an artery (i.e. body temperature and fluid flow shear stress). Briefly, the H-CSVSs were cut into 1 cm long pieces after 6 cycles of modification as described above. Each specimen was subjected to a tangential shaking wash in 25 ml of PBS at 37 °C under 80 rpm shaking conditions for up to 12 weeks. After each time point, 5 ml of washing solution was replaced with fresh PBS solution and the amount of released heparin was calculated using elemental analysis as mentioned above^36^.

### Blood compatibility

The antithrombogenic activities were evaluated by *in vitro* coagulation time tests including prothrombin time (PT) and activated partial thromboplastin time (APTT)^36^. Briefly, the 6 cycles modified tubes (n = 24, L = 1 cm) were subjected to tangential shaking wash in 100 ml of PBS at the same conditions mentioned in the previous section, followed by replacement of washing solution every week. At each time point, the specimens (n = 3) were removed and vigorously rinsed with PBS for three times. Each specimen was then incubated with fresh and healthy human blood plasma in a transparent plastic tube for 60 min at 37 °C. Afterward, 0.1 mL plasma solution was transferred to a transparent tube and incubated with reagents of each coagulation time test at 37 °C for 3 min. Finally, the clotting times were recorded as the interval between the addition of the CaCl_2_ solution (0.25 M) and formation of the clot by a photo-optical clot detection instrument (Coatron M1, TECO, Germany). The blood tests were carried out in accordance with the approved protocols of Shariati hospital and with the donor’s consent. The experimental protocols were approved by the Pasteur Institute of Iran.

Analysis of platelet adhesion was accomplished according to previously described^32, 36^. Briefly, platelet rich plasma (PRP) was obtained by centrifuging the human blood from a healthy volunteer at 850 rpm for 15 min at 4 °C. The specimens were immersed in PBS for 2 hours at 37 °C and placed in tubes containing 2 ml of fresh PRP (10^7^ cells/ml) and incubated in a humidified 5% CO_2_ atmosphere at 37 °C for 2 hours. After rinsing three times with PBS to remove loosely adherent platelets, the samples with adhered platelets were fixed in 4% glutaraldehyde solution at 37 °C for 12 h. The fixed samples were then dehydrated by exposure to a gradient of ethanol/distilled water mixtures (50%, 60%, 70%, 80%, 90%, and 100%), followed by drying in a fume hood overnight. Next, the specimens were sputter coated with gold and examined by SEM.

The hemolytic behavior of samples was examined according to previously described method^3^. Briefly, human red blood cells (HRBCs) were obtained by centrifugation of anticoagulated blood and then diluted with PBS to obtain HRBC suspension. Then, negative and positive controls were obtained by mixing 0.2 ml of diluted sample with 0.8 ml of PBS and water, respectively. Scaffolds were incubated in HRBCs suspension containing 0.2 mL of diluted HRBCs suspension and 0.8 mL of PBS for 2 hours at 37 °C followed by centrifugation at 2500 rpm for 5 min. Finally, the absorbance of the supernatant was examined by spectrophotometer at 540 nm. The hemolytic percentage (HP) was calculated according to the following equation:

7 $$\mathrm{HP}( \% )=(\frac{{D}_{t}-{D}_{nc}}{{D}_{pc}-{D}_{nc}})\times 100$$

where D_t_ is the absorbance of the test sample, D_nc_ and D_pc_ are the absorbance of negative and positive controls, respectively.

### Calcification behavior *in vivo*

The subcutaneous implantation model in Sprague–Dawley male rats was employed to study the calcification behavior of scaffolds *in vivo* ^36^. Briefly, the animals (140–150 g, obtained from the animal department of Pasteur Institute of Iran) were operated under 2% isoflurane mask anesthesia, after a 5% isoflurane induction. 12 rats were chosen and two parallel incisions were made in the middle back. Sheets of heparinized and non-heparinized scaffolds were symmetrically implanted for a periods of 6 and 12 weeks (n = 6 for each group). All the animal procedures were done in accordance with the approved animal protocol of Pasteur Institute of Iran and approved by the ethic committee of the same institute. On the experimental points, the animals were sacrificed and the samples retrieved and dried in a desiccator oven followed by disintegration with HNO_3_ (9 mL, 0.75 mol/L) and H_2_O_2_ (1 ml, 35%) at 75 °C for 16 hours. After centrifugation for 15 min at 3000 rpm, the supernatant was removed and the calcium content was determined using SPECTRO ARCOS ICP–OES (SPECTRO Analytical Instruments, Kleve, Germany).

### Histological analysis

In order to examine the inflammatory reactions for implanted scaffolds, the same procedure as mentioned in calcification section was performed on five separate Sprague–Dawley male rats. The entire subcutaneous tissue containing scaffolds were excised from euthanized animals after 6 weeks of implantation and fixed in 4% (w/v) buffered formalin overnight. Samples were processed, sectioned to 5 μm thickness and stained with hematoxylin and eosin (H&E) for histological analysis. The slides were observed using light microscope (Olympus BX41, Japan) and photographed by DP71 camera (Olympus, Japan).

### *In vitro* cytocompatibility assay

Human Umbilical Vein Endothelial cells (HUVEC; National Cell Bank of Iran) were used to evaluate the cytocompatibility of vascular grafts. The HUVECs were cultured in Ham’s F12 + DMEM (1:1) medium supplemented with 10% fetal bovine serum (FBS) and 1% penicillin/streptomycin solution (all culture reagents from Thermo Fisher Scientific, Gibco™). The medium was replaced every 2 days and the culture was incubated at 37 °C in a humidified atmosphere of 5% CO_2_. The cultures were periodically subcultured upon 70% confluency. Tubular scaffolds were cut longitudinally and the resulting sheets were punched into circular discs suitably sized for 96-well cell culture plates. The scaffolds were sterilized by immersion in 70% ethanol for 12 hours followed by three washes in sterile PBS for 20 min. After pre-incubation in complete culture medium at 37 °C overnight, the scaffolds were transferred to non-treated 96-well culture and seeded with the HUVE cells at a density of 5 × 10^4^ cells per well. The samples were transferred to non-treated 24-well culture plates 4 h after cell seeding and 1 ml of complete culture medium was added to each well followed by the medium replacement every 2–3 days. After being cultured for 1, 3 and 7 days, the cell-scaffold constructs were immediately rinsed three times with PBS, fixed in 2.5% glutaraldehyde overnight at room temperature, rinsed with PBS, postfixed in 1% OsO_4_ for 2 hours and then dehydrated through exposure to a gradient of ethanol and allowed to air dry at 4 °C. After sputter coating with gold, the morphology of cells was observed with a field emission scanning electron microscope (FESEM, MIRA3 TESCAN).

The presence and proliferation of HUVECs on the scaffolds’ surface were monitored by confocal microscopy. Briefly, scaffolds on days 1, 3 and 6 after cell seeding were fixed in 2.5% glutaraldehyde (Sigma-Aldrich) for an hour, followed by PBS wash and staining with PI solution (Sigma-Aldrich) for 1 min. The samples were observed by Leica TCS SPE confocal microscope with excitation/emission at 532/615 nm. The images of the scaffolds were captured and stacked from the surface to depth of 85 µm with 5 µm increments.

MTT assay was also carried out to determine cell adhesion and proliferation on the scaffolds 1, 3, 5 and 7 days after cell seeding^50^. Briefly, 200 μl of cell suspension (10^5^ cells/ml) were cultured on scaffolds in non-treated 96-well culture plates. The medium was removed and 110 μl of serum free culture medium containing 0.5 mg/ml 3-(4,5-dimethylthiazol-2-yl)-2,5-diphenyltetrazolium bromide (MTT) were added and incubated at 37 °C and 5% CO_2_ for 4 h. Samples were then transferred to 2 ml tubes and centrifuged at 1000 rpm for 5 min. The supernatant was aspirated and replaced with 110 μl of dimethyl sulfoxide (DMSO) and shaken vigorously for 5 min. The tubes were centrifuged again at 12000 rpm for 10 min and 100 μl of the supernatant of each sample was aspirated into a new 96-well plate, and the absorbance subsequently measured at 570 nm using a microplate reader (Synergy HTX, Biotek). Non-seeded scaffolds were used as negative controls for each time point. The methods were carried out in accordance with the approved protocol of National Cell Bank, Pasteur Institute of Iran. It should be mentioned that for all the experiments involving human subjects (cellular and blood tests), written informed consent was obtained.

Furthermore, A7r5 cells were used to reveal that heparin loading method in the current work would not exert detrimental effects on smooth muscle cells of outer sponge layer. To this end, outer sponge layer was detached from inner layer and A7r5 cells (10^5^ cells/ml) were cultured in DMEM medium supplemented with 10% fetal bovine serum (FBS) and 1% penicillin/streptomycin solution. The medium was replaced every 2 days and the cells were incubated at 37 °C in a humidified atmosphere of 5% CO_2_. MTT assay on days 1, 3, 5 and 7 post cell seeding was used to show the proliferation of cells.

### Statistical analysis

Data were statistically analyzed using SPSS v. 18.0 software. Pair groups were analyzed with the Student’s t-test. Multiple samples were evaluated by one-way ANOVA followed by Student–Newman–Keuls post hoc tests. Measures are expressed as means ± standard deviations and considered significant at *p* < 0.05.

## Conclusions

Herein, a composite tubular scaffold comprising concentric silk fiber reinforced SF sponge and SF sponge layers was designed and characterized in order to evaluate its potential use as a small-diameter vascular graft. The present construct simultaneously provided all the biomechanical properties (flexibility, suture retention strength, burst pressure, and compliance) in the desirable range compared to previous silk-based vascular grafts. The employed method provided a dimorphic structure with a quite smooth heparinized lumen and highly porous external layer. However, the obtained results can be further enhanced by optimizing the yarn number and diameter. Furthermore, our findings revealed suitable sustained antithrombogenicity, cytocompatibility and nonhemolytic attributes of H-CSVS. Accordingly, it could be considered as an improved alternative for small-diameter vascular grafts. The combination of required features in the same construct confirms that H-CSVS represents a reliable and promising graft for future clinical translation of tissue engineering of vascular grafts. Considering these promising results, integration of growth factors and then *in vivo* investigations in a large animal model are in progress.
